# Supplementary material for: Fusion of single-cell transcriptome and DNA-binding data, for genomic network inference in cortical development
Source: BMC Bioinformatics. 2021 Jun 4;22:301. doi: 10.1186/s12859-021-04201-9 (PMC8176738; doi:10.1186/s12859-021-04201-9)
Supplement: Supplementary file 1 — Additional file 1. Supplementary Tables and Supplementary Information. [file 12859_2021_4201_MOESM1_ESM.pdf]

Fusion of single-cell transcriptome and DNA-binding data, for genomic network inference in cortical development

Thomas Bartlett - University College London

Appendix

Supplementary Tables

vRG (stem cell)	oRG (stem cell)	IPC	Inhibitory neuron	Excitatory neuron
CDON	ABAT	PPP1R17	DLX1	ENC1
FBXO32	CDCA7L		DLX2	GRIA2
LDHA	CDK6		DLX5	KCNQ3
NAMPT	ETV1		DLX6-AS1	LRP8
NAPEPLD	ETV5		ERBB4	MLLT3
PALLD	FABP7		FAM65B	MYT1L
SALL1	FAM107A		PLS3	NEUROD6
SHISA2	IDI1		PRKCA	NRP1
TMEM47	IL6ST		DLX2	PALMD
	LIFR		DLX6-AS1	PLS3
	MOB3B		ERBB4	PLXNA2
	MOXD1		GAD1	POU3F2
	NRG1		MAF	PPP2R2B
	PTPRZ1		NFIA	QKI
	SAT1		NFIB	SATB2
	SEL1L3		NRXN3	SEMA3C
	SEMA5A		PDZRN4	SLA
	SLITRK2		RUNX1T1	SLC24A2
	TCF7L2			SORBS2
	TMEM132B			SOX2-OT
	TNC			SRGAP1
				STMN2
				TTC28
				ZFP36L1
				ZNF238

Table S1: *Marker genes used to generate color-intensities (according to mean expression) in Fig. 1.*

RELN	RELN->TBR1->CTIP2->CUX1+SATB2 are activated sequentially, for neurons progressively further from the ventricle to pia
CUX1	determines layer II, layer III, layer V callosal projection neurons
SATB2	marks maturing excitatory neurons, determines layer II, layer III, layer V calosal projection neurons
ASCL1	marks cortical projection neuron progenitors
SOX6	marks progenitors of glutamatergic projection neurons in the cortex
PAX6	marks progenitors of glutamatergic projection neurons in the cortex, specifies regional, laminar and neurotransmitter fates
SP8	marks progenitors of glutamatergic projection neurons in the cortex
ADCY1	maturing excitatory neurons
EMX2	marker of lower-layer neurons
SOX2	marks lower-layer neurons
RELN	layer I neurons
CUX2	determines and marks layer II, layer III, layer V calosal projection neurons
TBR1	determines layer I neurons, marks somatosensory cortical pyramidal cells, layer V-VI, and sub-cortical projection neurons
LHX2	marks layer II, layer III, layer V callosal projection neurons
EPHA3	marks layer II, layer III, layer V callosal projection neurons
FEZF2	determines and marks layer V projection neurons
OTX1	marks layer V-VI projection neurons
SOX5	marks layer V projection neurons, determines and marks layer VI projection neurons
TLE1	marks layer VI-V projection neurons
FOXP2	marks layer VI projection neurons
RASGRF2	marks somatosensory cortical pyramidal cells, layer II-III
CUX2	marks somatosensory cortical pyramidal cells, layer II-III
RORB	marks somatosensory cortical pyramidal cells, layer IV-V
PLCXD2	marks somatosensory cortical pyramidal cells, layer IV-V
THSD7A	marks somatosensory cortical pyramidal cells, layer V
FOXP2	marks somatosensory cortical pyramidal cells, layer VI
SYT6	marks somatosensory cortical pyramidal cells, layer VI
NR4A2	marks claustrum pyramidal cells
NEUROG2	marks layer II/III pyramidal neurons
TLE3	marks layer II-III
MDGA1	marks layer II-III
CYP39A1	marks layer IV
DTX4	marks layers II-IV
MARCKSL1	marks layers II/III and Vb
FOXO1	marks layer Vb
LIX1	marks layer Vb
OMA1	marks layer Vb
LDB2	marks layer Vb
CRIM1	marks layer Vb
IGFBP4	marks layers II-III and Vb-VI
DKK3	marks layers Vb-VI
TLE4	marks layers Vb-VI
SEMA3E	marks layers Vb-VI
NR4A3	marks layer VI
LXN	marks layer VI
ID2	marks layers II/III and Vb-VI
LMO3	marks layers II-III and VI

Table S2: *A selection of genes important for excitatory neuronal identity.*

Supplementary Information

List of genes inferred as regulated by the TFs shown in Figure 6

TCF4: AMZ2P1, AP1S2, ARHGDIA, ATXN1L, BCL11B, C19orf25, C6orf106, CALCOCO1, CAMK2B, CBFA2T2, CBLB, CCDC88C, CELF3, CHRDL1, CLEC16A, CLSTN3, CPSF4, DOCK4, DUOX1, EEF2, FAM110A, FAM126A, FAM171A2, FBLN1, FTL, FZD7, GAK, GCC1, GNG3, GPM6A, GSE1, HADHA, HDAC2, HECTD4, HID1, HPCAL1, IGDCC3, IGSF3, ITPR2, KALRN, KCNQ3, KCTD13, KIAA1324, LIN7B, LINC00476, LRP8, MAP6, MARCH4, MEX3A, MN1, MXI1, NAA35, NAB1, NCAN, NCOR2, NCS1, NDUFB11, NEUROD2, NFASC, NHLH1, NRP1, PALMD, PGAP1, PLA2G12A, POLB, PPP2R1B, PRRC2B, PSD2, PTPRS, PUS3, R3HDM2, RAB3A, RBCK1, RBFOX2, RPL10, RPL37A, RPS6KB2, SCAF1, SERGEF, SERINC5, SEZ6, SH2D3C, SIDT2, SLC25A38, SLC38A1, SLC52A2, SLCO5A1, SMARCD3, SNAP25, SORBS1, SRRM4, ST8SIA2, STMN1, SUPV3L1, TBCE, TES, TMEM169, TMEM74, TMEM86A, TMSB10, TRIOBP, TSPAN13, TTC9B, TUBB3, UNC5A, WDR20, WNK2, YWHAG, ZDHHC20, ZFP30, ZNF286A, ZNF286B, ZNF333, ZNF423, ZNF512B, ZNF660, ZYG11B. NEUROD2: ACTL6B, ADCYAP1R1, ADRA2A, APBB1, ATP11C, ATP9A, C11orf87, CACNA1B, CALCOCO1, CDH12, CECR2, CERS6, CHRDL1, CLMP, CNTN2, CNTNAP2, COMMD9, CPSF4, DANCR, DOCK4, DOK4, DPY19L1, ENC1, EXOSC4, FABP7, FAM110A, FAM126A, FAM13A, FAXC, FBLN1, FGF13, GNG3, GNG4, GRIA3, HDAC2, HECTD4, HSPBP1, IGDCC3, IMP4, ITPR2, KIAA1324, KIDINS220, L1CAM, LAMC2, LRP8, MAP2, MAPK8, MBTD1, MEX3A, MFSD6, NCS1, NDRG1, NEDD4L, NFASC, NTM, PAG1, PAK1, PCDHB2, PCDHB5, PDHA1, PFDN4, PGAP1, PHC2, PMEPA1, POU3F1, PP-FIA2, PPP1R14B, PPP1R17, PRKG2, RBCK1, RBFOX1, RBFOX2, RPL37A, RTN2, RTN4, SATB2, SCAF1, SEPT3, SEZ6, SEZ6L2, SHB, SIDT2, SLC22A23, SNAP25, TMEM158, TMEM86A, TRIOBP, YKT6, YWHAG, ZDHHC20, ZNF142, ZNF266, ZNF574, ZNF616, ZNRF1. HIC2: AD-CYAP1R1, AMZ2P1, APBB1, ARHGDIA, ARIH2, ATP11C, BANP, C10orf88, CACFD1, CBFA2T2, CBLB, CEP170, CHRDL1, CMPK1, DISP2, DOCK4, DPY19L1, ELMO1, EPHA3, FAM13A, FGF13, GRIA3, HDAC2, HEATR3, HES6, HPCAL1, HS6ST1, IGDCC3, IGFBPL1, INPP5K, IRS1, KCNQ3, KIAA1324, KIDINS220, L1CAM, LMBR1L, MAPK8IP3, MRPL54, NARF, NCAN, NDRG1, NDUFB11, NDUFB7, NFASC, NFIA, NKAIN1, OCIAD2, PCDHB10, PHF21B, POLB, PPP1R14B, PPP2R1B, PTPRS, R3HDM2, RBCK1, RPL10, SCN3A, SEC14L2, SERINC5, SEZ6, SIDT2, SLC11A2, SLC25A38, SLCO5A1, SMARCD3, SORBS1, SORL1, SOS1, STK17A, SULT4A1, TMSB10, TPRG1L, TRIM62, TRIOBP, UPK2, WDR20, ZDHHC20, ZDHHC8P1, ZNF266, ZNF616, ZNF704, ZNRF1. NFIX: ACSF3, ADCYAP1R1, ADM2, ADRA2A, ARHGDIA, AT-CAY, C19orf25, CACFD1, CACNA1B, CBFA2T2, CBLB, CECR2, CHRNA4, CNTNAP2, CRMP1, DANCR, DISP2, DOPEY2, DYNC1H1, EEF2, FAM110A, FAM126A, FAM13A, GCC1, GRIK5, GSE1, GTF2H5, HCFC1R1, HES6, HPCAL1, INPP5K, KCTD16, L1CAM, LRP8, MARCKSL1, MAX, MRPL54, NARF, NDUFB7, NPTX2, NRP1, OCIAD2, PCDHB10, PEX11B, PPP1R14B, PPP2R2B, PRKG2, RAB12, RPL37A, RUNDC3A, SATB2, SCN3A, SEPT3, SEZ6, SLC25A38, SMURF1, SORL1, STK17A, STK31, TBCE, TRIM62, USP3, WDR20, ZDHHC8P1, ZNF142, ZNF266, ZNF286A, ZNF286B, ZNF397, ZNF660, ZYG11B. MEIS3: ANK3, AP1S2, ARHGEF3, ARIH2, ATG13, ATP1A3, BRD3, CACFD1, CALCOCO1, CECR2, CEP170, CHGA, CLMP, COMMD9, ELMO1, EXOSC4, FABP7, FAHD1, FAM220A, FGF13, GAK, GPD1L, IGFBPL1, ITPR2, L1CAM, NAA35, NAB1, NARF, NCKAP5L, NDRG1, NHLH1, PAK1, PCDHB5, PEX11B, PHC2, PHF21B, PHLDA1, PLA2G12A, PLXNA2, POLB, PPP2R1B, PPP2R2B, PSD2, PSMB7, PTPRS, RBFOX2, RPL31, RUNDC3A, SARM1, SENP6, SERINC5, SIDT2, SLC52A2, SLCO5A1, SMARCD3, SMURF1, SORL1, STK17A, SULT4A1, TBCE, TES, TMEM74, YWHAG, ZDHHC8, ZDHHC8P1, ZNF266, ZNF333, ZNF512B, ZSCAN20. TCF3: AD-CYAP1R1, ADRA2A, ANKRD52, ATG13, BCL11B, BRD3, CACFD1, CALCOCO1, CAMK2B, CDK2AP1, CERS4, CLEC16A, CNTN2, DPY19L1, EEF2, GNG4, HS6ST1, ITPR2, KCNQ3, KIAA1324, KIDINS220, L1CAM, LIN7B, LYPD6, MN1, MYT1, NDRG1, NDUFB11, NDUFB7, NFASC, PAK1, PPP2R1B, PREX1, PTPN4, RAB3A, RBFOX2, RPL31, RUNDC3A, SERINC2, SH2D3C, SIDT2, SLC25A38, SLCO5A1, SOS1, TMEM169, TMEM74, TMEM86A, UNC5A, ZDHHC20, ZFP30, ZNF333, ZNF423. TEAD1: ACTL6B, AP1AR, APBB1, BANP, BRD3, C6orf106, CDH12, CEP170, DEPDC7, DOCK4, EPHA3, F2R, FABP7, FAM126A, GSE1, HADHA, HDAC2, IGFBPL1, IMP4, KIAA1324, LINC00476, MFSD6, NAB1, NDRG1, NEDD4L, NHLH1, PDHA1, PEX11B, PFDN4, PHF21B, PHLDA1, PPP1R17, RAB12, RPL31, RTN1, RTN4, SLCO5A1, SNHG8, SOS1, SRRM4, SULT4A1, TMEM74, TTC9B, UBXN7, USP3, ZNF217, ZNF266, ZNF333. JUND: ACSF3, ARHGDIA, ARRB1, ATG13, ATXN1L, BRD3, CERS6, CLMP, DOCK4, DPY19L1, DYNC1H1, F2R, FAXC, FZD7, GALNT11, GNG4, H3F3B, HDAC2, HPCAL1, IMP4, KCNQ3, KIF21B, MYT1L, NCS1, NDRG1, NRN1, PDHA1, PFDN4, PGAP1, PRDM8, PSMB7, RPL10, SETD7, SMARCD3, SNAP25, SNHG8, SOGA3, SORBS1, SOS1, SULT4A1, TES, TTC9B, USP3, VASH1, VPS37B, VPS37D, ZNF704. LHX2: AKT3, ANKRD52, AUTS2, CBLB, CLVS1, CNR1, COL9A2, CTNNBIP1, EPHA3, FAM149A, FAXC, FBLN1, FOXP4, GPM6A, HECTD4, HMG20B, HS6ST1, JARID2, KIAA1456, KLHL23, KRR1, LHFPL4, LRP8, MAPK8, MARCKS, MLLT3, NAA35, PCDHB5, PCLO, PHF21B, PPFIA2, PRDM8, RASGEF1B, SATB2, SENP6, SEZ6, SHB, SLC24A2, SLC4A7, SORBS1, SSTR2, SUPV3L1, SYT1, TRIOBP, UNC5A, ZNRF1. ZNF384: BSN, CALCOCO1, CBFA2T2, CCNJ, CHRNA4, CNTNAP2, DANCR, DOK4, FABP7, GTF2H5, HADHA, KALRN, KCNQ3, KLHL26, MPP1, NAB1, NARF, NCKAP5L, NDRG1, OCIAD2, PAK1, PCDHB5, PCLO, PDE1C, PHF21B, PTPN4, RBFOX2, RPL37A, RTN4, SERINC2, SERINC5, SMARCD3, SORBS1, SYT7, ZNF286A. THAP1: AMZ2P1, ARFGAP1, BRD3, CACNA1B, CALCOCO1, CBFA2T2, CLMP, DEPDC7, DOK4, DPY19L1, EPHA3, EXOSC4, GALNT11, GRIK5, HEATR3, IGDCC3, KCNQ3, LIN7B, MAX, MPP1, NARF, NTM, PAG1, PCDHB2, PEX11B, PHC2, POLB, SNAP25, SORL1, TMEM158, UPK2, VPS41, ZNF574. MEIS1: ADCYAP1R1, ATP11C, BHLHE22, BRD3, C10orf88, CHRDL1, DOPEY2, GNG3, IGDCC3, NDUFB11, NDUFB7, NHLH1, NPTX2, PAK1, PHC2, PPP2R1B, PPP2R2B, QSOX1, RAB12, RP9P, RUNDC3A, SARM1, SEMA5B, SEZ6, SLCO5A1, SNAP25, SORBS2, UNC13B, ZNF266, ZNF488, ZNF616, ZNF778. MGA: AMZ2P1, ANKRD52, CALCOCO1, CHN2, CLEC16A, CLMP, DPY19L1, FAHD1, FAM13A, FAM171A1, FAXC, GMEB2, HIVEP3, HS6ST1, KIAA1324, KLHL26, NFIA, PARP16, PPP2R2B, PTPRA, PTPRS, R3HDM2, RAB12, RUNDC3A, SLA, SNAP25, SORL1, SRRM4, SYT4, TRIOBP, YWHAG, ZNF217. MEIS2: ADCYAP1R1, ARRB1, BRD3, CLMP, CRMP1, ELMO1, FAM110A, FAM220A, FGF13, GPD1L, GPM6A, GTF2H5, H3F3B, LAMC2, MAPK8, NAB1, NCKAP5L, NHSL1, PAK1, PHF21B, PTPRS, R3HDM2, RBCK1, SATB2, SORL1, SULT4A1, TMEM120A, TMEM86A, TMSB10, USP3, ZNF512B, ZYG11B. STAT3: AKT3, ATP11C, CDH12, CMPK1, DISP2, DOPEY2, DUSP14, GNG4, KALRN, KIAA1324, KIF21B, NCKAP5L, OCIAD2, PCDHB2, PCLO, PDHA1, PEX11B, PHF21B, PHLDA1, PPP2R2B, R3HDM2, SLC25A38, SNAP25, SORBS2, SRRM4, STK31, ZNF266, ZNF333, ZNF616, ZSCAN20.
